# Can, Want and Try: Parents’ Viewpoints Regarding the Participation of Their Child with an Acquired Brain Injury

**DOI:** 10.1371/journal.pone.0157951

**Published:** 2016-07-01

**Authors:** Melanie Thompson, Catherine Elliott, Claire Willis, Roslyn Ward, Marita Falkmer, Torbjӧrn Falkmer, Anna Gubbay, Sonya Girdler

**Affiliations:** 1 School of Occupational Therapy and Social Work, Curtin University, Perth, Western Australia, Australia; 2 School of Sport Science, Exercise and Health, University of Western Australia, Perth, Western Australia, Australia; 3 School of Paediatrics and Child Health, University of Western Australia, Perth, Western Australia, Australia; University of Florida, UNITED STATES

## Abstract

**Background:**

Acquired brain injury (ABI) is a leading cause of permanent disability, currently affecting 20,000 Australian children. Community participation is essential for childhood development and enjoyment, yet children with ABI can often experience barriers to participation. The factors which act as barriers and facilitators to community participation for children with an ABI are not well understood.

**Aim:**

To identify the viewpoints of parents of children with an ABI, regarding the barriers and facilitators most pertinent to community participation for their child.

**Methods:**

Using Q-method, 41 parents of children with moderate/severe ABI sorted 37 statements regarding barriers and facilitators to community participation. Factor analysis identified three viewpoints.

**Results:**

This study identified three distinct viewpoints, with the perceived ability to participate decreasing with a stepwise trend from parents who felt their child and family “can” participate in viewpoint one, to “want” in viewpoint two and “try” in viewpoint three.

**Conclusions:**

Findings indicated good participation outcomes for most children and families, however some families who were motivated to participate experienced significant barriers. The most significant facilitators included child motivation, supportive relationships from immediate family and friends, and supportive community attitudes. The lack of supportive relationships and attitudes was perceived as a fundamental barrier to community participation.

**Significance:**

This research begins to address the paucity of information regarding those factors that impact upon the participation of children with an ABI in Australia. Findings have implications for therapists, service providers and community organisations.

## Introduction

Acquired brain injury (ABI) is a leading cause of permanent disability among children worldwide [1, 2]. In Australia, approximately 20,000 children under the age of 15 years are currently living with an ABI, a figure which is increasing annually [3, 4]. Each ABI is unique and is influenced by the child’s developmental stage at the time of injury and the severity of the cause [5, 6]. Recovery from ABI is unpredictable; depending on the severity, children may regain their pre-injury skills or experience long-term physical, cognitive or behavioural impairments [6, 7]. Children with moderate to severe ABI (diagnosed according to immediate medical outcomes following injury) are likely to have difficulty with independence in everyday tasks [3, 8, 9]. These persisting issues can significantly impact their participation in everyday life [3, 8].

Participation is vital for development during childhood [10]. Participation in the community (typically leisure and recreation) provides children with the opportunity to explore their own personal interests, grow as individuals and most importantly; to enjoy life [11–14]. Understanding outcomes related to participation is especially important when considering the impact of complex diagnoses like ABI, as they focus on the children’s involvement in life situations rather than on their impairments [6, 14]. However, children with moderate to severe ABI experience significant participation restrictions when engaging in social and community based leisure activities [6, 15]. As a result, children with an ABI in Australia participate in more activities in the home environment with family members than with friends in the broader community [6].

Literature examining the barriers and facilitators to participation in children with ABI is limited [6]. However, due to the similarities in participation restrictions faced by children with ABI and children with neurodevelopmental disabilities, it is likely that they face similar barriers and facilitators. The International Classification of Functioning: Children and Youth Version (ICF-CY) provides a holistic framework to conceptualise the consequence of childhood ABI on participation [14]. The ICF-CY captures the dynamic interaction between health conditions (impairments), a child’s activity (carrying out an action or completing a task) and participation (involvement in life situations) in their ‘context’, inclusive of environmental and personal factors [14].

Within the ICF-CY, ‘facilitators’ support a child’s participation, while ‘barriers’ may cause participation restrictions [16]. For example, within the immediate family, parents can act as primary facilitators to their child’s participation by actively searching for opportunities, making their time available, and committing to providing individualised support [6, 17, 18]. Conversely, parents may inadvertently act as barriers to participation as they struggle to prioritise their competing demands of home, work, family and their own health [17]. Similarly, while friendships can motivate a child with a disability to participate in physical activity, sports and general play [17], a lack of friendships or difficulty making friends due to communication impairments, may act as a barrier to community participation [6]. Other family-dependent environmental factors, such as physical geography and socio-economic status have been identified as possible barriers or facilitators to participation for children with disability [18, 19].

Societal attitudes have been identified as a significant barrier to participation in physical activities for children with a disability [17]. Furthermore, the ‘invisible’ nature of ABI can lead to a lack of understanding from peers and professionals, contributing to participation restrictions [6]. While most services, systems and policies aim to be inclusive, there is a lack of services that truly facilitate community participation for children with disability [17]. This problem is often compounded by mainstream programs that are unwilling or unable to be inclusive [17]. Strong facilitators can include peers and community activity organisers who are accepting of children with ABI, and when families embrace the value of community participation embedded in many cultures, including Australian [19, 20].

The participation outcomes for children following an ABI are not well understood [6, 21–23]. While the abovementioned studies identify barriers and facilitators to participation for children with neurodevelopmental disabilities, there is still a need to determine their relative pertinence to ABI alone. Therefore, the aim of this study was to identify *viewpoints* of parents regarding *what* barriers and facilitators are *most pertinent* to the community participation of their child with an ABI.

## Materials and Methods

**Fig 1:**
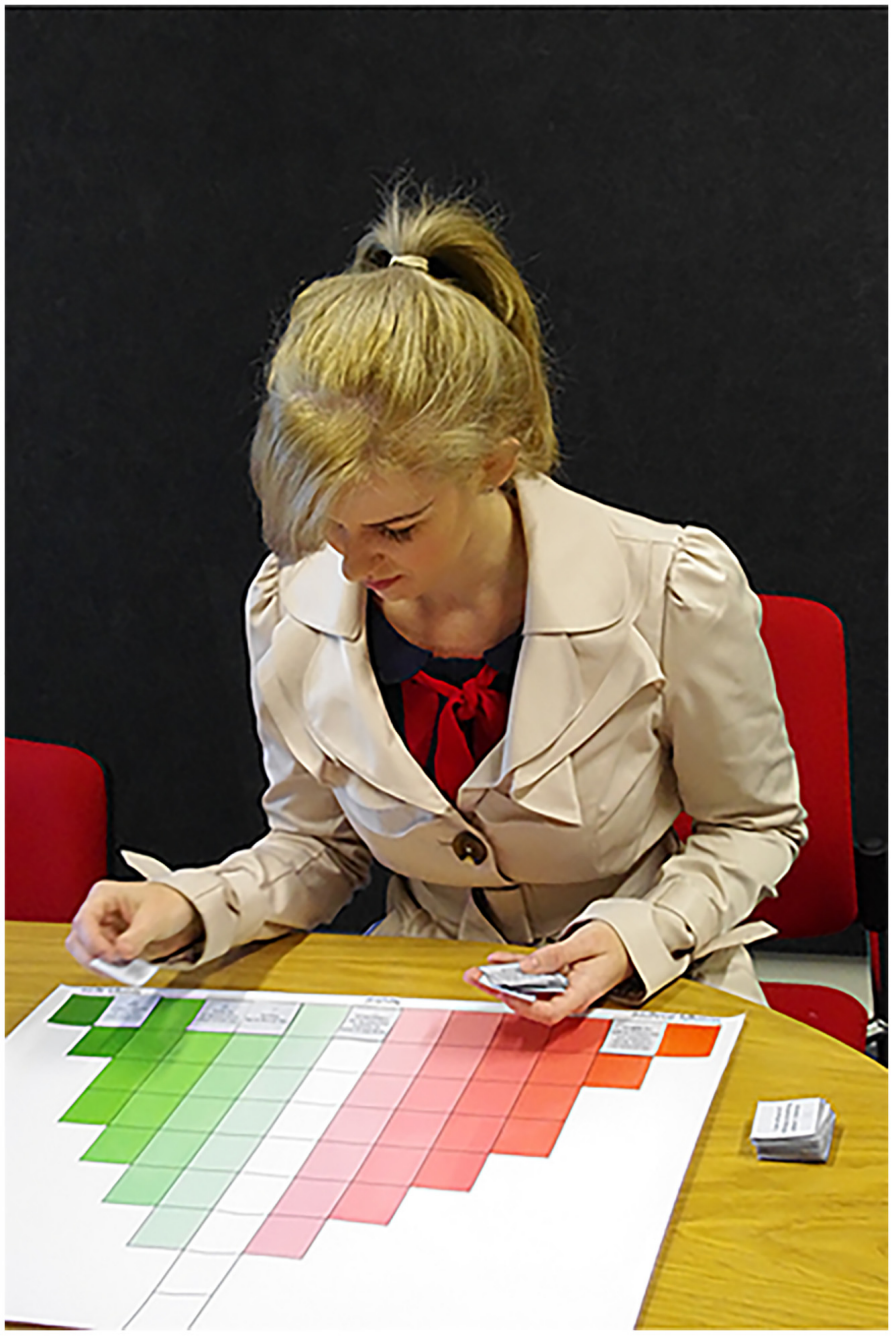
Q sort Task being Undertaken.

The Q-method was used to identify the viewpoints of the participants [24]. The Q-method allows for exploration of experiences by asking each individual to conduct a Q-sort- sorting a set of predetermined statements onto a normally distributed grid (Fig 1) ranked from strongly agree, to strongly disagree. The completed Q-sort provides an overview of the participants’ viewpoints on the topic in question [24–26]. The fact that ‘the grid’ comprises only as many possible positions as there are statements allows for a discrimination of viewpoints that may not be identifiable by simply identifying items in a questionnaire or through interviews [25, 27]. The Q-method employs both inductive and deductive approaches which enables an in-depth understanding of an array of participants perspectives and beliefs with regards to a particular topic of interest [24, 27, 28]. Q-method does not require large numbers of participants as its goal is to identify the preferred viewpoints of a defined group, with between 40–60 participants considered sufficient to meet this aim [27, 29]. The Q-method involves five steps as described below: 1) developing the Q-concourse, 2) identification of statements, 3) administration of the Q-sort, 4) data analysis and 5) interpretation of factors in the context of the current study [25, 27, 30].

**Fig 2:**
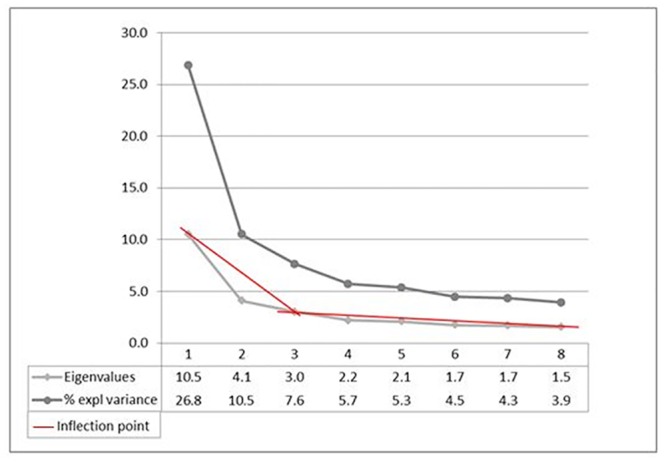
Scree Plot Demonstrating the Inclusion of Three Factors.

1. The Q-concourse for the current study was developed through a systematic review focused on identifying barriers and facilitators impacting on participation of Australian children with neurological impairments and a systematic review identifying elements that contribute to meaningful participation for children and youth with disabilities [24, 31].
2. Based on the concourse, 50 statements were identified and reviewed by an advisory steering group [32], developed to guide and direct a larger project that this study was designed to inform. The group included; an adolescent with an ABI, parents of children and adolescents with an ABI, clinicians working in hospital and community settings, a disability policy advisor, and representatives from non-government organisations and community support centres. Following feedback from this group, the 50 statements were reduced to 37 statements (Table 1) and many wording changes were made. Overall, 62% of the original statements were changed on advice from the steering group. A speech pathologist reviewed the statements for language errors and the final set of statements was piloted with three clinicians from the ABI team at a children’s hospital; a clinical psychologist, a clinical nurse specialist and an occupational therapist.
3. The Q-sort was distributed to 41 participants recruited through convenience sampling whilst waiting to attend their child’s routine ABI clinic appointment at a paediatric hospital in Western Australia. Inclusion criteria were; parents of children aged 5–17 with an ABI at least three months post-injury, and an ABI diagnosed as moderate/severe by a paediatrician. Participants were given verbal instructions from a script to ensure consistency and inter-rater reliability of the researchers. After written consent, the participants were asked to sort the statements onto the ‘grid’ (see Fig 1) according to a eleven-point continuum ranging from; +5 ‘strongly agree’ to 0 for the ‘neutral’ column, and -5 for ‘strongly disagree’ [24, 25], in order of perceived relative priority [27]. The participants were reminded that there were no right or wrong places in regards to sorting the statements and that the completed Q-sort would provide a snapshot of the participants’ experience of the barriers and facilitators to their child’s participation at that point in time [33]. Upon completion of the Q-sort, parents were asked if they perceived any statements irrelevant or missing; and a satisfaction score was obtained in relation to the extent to which the participant felt that their final sort represented their viewpoint [27, 34]. Data was collected on length of time to complete the Q-sort and the participant’s relationship to the child. Additionally, data was gathered to complete an Index of Relative Socio-economic Disadvantage [32], to classify the participants as residing in remote, outer regional or metropolitan areas [33].
4. Each participant’s pattern of statements (Q-sort arrangement) was manually entered into the PQ Method program [35], in which a per-person factor analysis was conducted, in order to identify the most prominent viewpoints. Five consecutively fulfilled criteria were used in order to select factors [36]. They consisted of: 1) the “magic number seven” which is the default minimum number of factor extraction in PQ Method, 2) the Kaiser-Guttman Criterion, which allows factors with an eigenvalue greater than 1.0 to be retained [37, 38], 3) ‘Two significantly loading sorts’ states that at least two significant factor loadings are required per retained factor, 4) Humphrey’s rule, which states that when multiplied, the sum of the two significant factors with the highest (positive and/or negative) rankings must be greater than twice the standard error and 5) the ‘Scree test’, which indicates that factors displayed prior to the scree plot ‘levelling out’ should be retained (see Fig 2) [39]. These five criteria justified the inclusion of three factors for further data analysis and interpretation. Varimax rotation was run on the three factors, determining the best solution for the explained variance, and the individual sorts which significantly (p<0.05) loaded onto a factor were then uncovered [39]. This process ensured that participants did not load onto more than one factor, thus revealing the distinct viewpoints among the participants [36].
5. Interpretation of results. Data interpretation commenced with naming the three factors (which are henceforth synonymous with ‘viewpoints’). This was a collaborative process including four clinicians in the field of ABI and participation. Discussion regarding each viewpoint’s defining statements was held until consensus was reached and each viewpoint was named.

**Table 1:** Q-set Statements, Factor arrays of viewpoints (Ranking) and Z-scores, All Statements are Shown and Ordered According to Factor Loadings.

Statements	Factor array (Z score)			Consensus or Contended statement
	F1	F2	F3	
4. My child receives enough funding to cover their community activity needs	**-1 (-0.57)**	-4 (-1.42)	-3 (-1.10)	
5. I feel obliged to supervise my child in their community activities	0 (-0.14)*	2 (0.68)	3 (0.94)	
6. I have enough time to help my child participate in the community	2 (0.73)*	-2 (-0.89)	-1 (-0.57)	
10. My child finds it difficult to play with other children	-5 (-1.86)*	2 (0.57)	1 (0.47)	Contended statement
17. My child is proud of participating in community activities that are meaningful to him/her	**4 (1.22)**	4 (1.69)	5 (2.00)	Consensus statement
19. I feel discouraged when searching for community activities that are appropriate for my child	-3 (-1.22)*	1 (0.52)	2 (0.49)	
24. There are enough activity programs in the community that meet my child’s needs	0 (-0.10)*	-2 (-0.68)	-3 (-1.27)	
27. My child is easily able to participate in community activities	3 (1.15)*	-1 (-0.47)	-1 (-0.14)	
32. Health professionals have helped/encouraged my child to participate in the community	2 (0.77)*	0 (-0.25)	-1 (-0.11)	
33. My child finds it hard to participate in community because of their difficulty concentrating and/or paying attention	**-2 (-1.07)**	1 (0.56)	0 (0.14)	
34. It is hard for my child to participate in community activities because of behavioural difficulties	-2 (-1.13)*	0 (-0.10)	1 (0.30)	
3. My stress levels influence how much I am able to help my child participate in community activities	0 (0.03)	3 (1.29)*	1 (0.40)	
14. I don’t need to travel long way to get to community activities that are suitable for my child	0 (0.26)	-2 (-0.58)*	0 (0.29)	
18. My child wants to do what his/her friends and/or siblings do in the community	3 (0.98)	4 (1.96)*	3 (1.12)	
21. It is not difficult to get my child to and from community activities	1 (0.54)	-3 (-1.40)*	2 (0.64)	Contended statement
37. My family is motivated to participate in the community	1 (0.66)	-1 **(-0.56)**	0 (0.15)	
9. Adults supervising activities in the community make an effort to include my child	2 (0.77)	2 (0.88)	0 (-0.05)*	
13. Mainstream community activity programs include my child	0 (0.37)	0 (0.21)	-2 (-0.60)*	
29. My child has enough energy to complete community activities	3 (0.96)	2 (0.78)	-1 (-0.40)*	
30. My child finds it hard to participate in the community because of mobility difficulties	-2 (-1.00)	-2 (-1.12)	3 (1.12)*	Contended statement
12. People in the community understand my child’s ABI, they are welcoming and know how to interact with my child	1 (0.51)*	-1 (-0.37)*	-5 (-2.61)*	Contended statement
11. My child has friends in and out of school	5 (2.04)*	-1 (-0.31)*	2 (0.87)*	Contended statement
2. When necessary I educate supervising adults on how to include my child	**-1 (-0.20)**	**3 (1.04)**	**1 (0.39)**	
28. My child’s school organises out-of-school events that are inclusive	**-1 (-0.60)**	**0 (-0.16)**	-4 (-2.28)*	Contended statement
22. Community activities suitable for my child are within my family’s budget	**1 (0.54)**	-3 (-1.26)*	**-1 (-0.11)**	
25. It makes me happy when my child enjoys participating in community activities	4 (1.90)	5 (2.29)	4 (1.71)	Consensus statement
36. My child is motivated to participate in activities in the community	2 (0.88)	3 (0.92)	0 (0.24)	Consensus statement
31. My child finds it hard to participate in the community because of communication difficulties	-4 (-1.60)	1 (0.35)	0 (0.14)	Contended statement
1. I actively seek opportunities for my child to participate in the community	1 (0.66)	1 (0.44)	1 (0.47)	Consensus statement
16. My child does not enjoy learning new skills and/or is not persistent with learning new skills	-3 (-1.27)	-3 (-1.17)	-2 (-0.67)	Consensus statement
15. I feel less isolated when my child is involved in community activities	0 (0.19)	1 (0.52)	0 (0.11)	Consensus statement
26. Community activities suitable for my child have long wait lists	-1 (-0.59)	-1 (-0.26)	-2 (-0.64)	Consensus statement
8. My child gets frustrated when he/she is not able to do what is required when participating in community activities	**-1 (-0.49)**	0 (0.02)	4 (1.20)*	
20. It is not important for my child to participate in the community	-4 (-1.77)	-4 (-1.44)	-2 (-0.76)	
35. My child doesn’t want to participate in community activities	-3 (-1.26)	-5 (-2.06)	-3 (-0.83)	
7. My child has a role model that inspires/encourages them to participate in community activities	0 (0.32)	0 (-0.018)	2 (0.79)	
23. Community activities suitable for my child are advertised widely enough	-2 (-0.62)	0 (-0.21)	-4 (-1.81)*	

Note: Bold represents distinguishing statements at p<0.05 and * represents distinguishing statements at p<0.01.1)

### Ethical considerations

The procedures of this study conformed to the Declaration of Helsinki [29], and ethical approval was received from Curtin University, Perth, Western Australia (HR01/2014), The University of Western Australia Perth, Australia (RA/4/1/6556), and Princess Margaret Hospital (2013099). Participants were provided with an information sheet explaining the aim of the project, how data was to be confidentially stored and de-identified in any presentation of results. Furthermore, participants were reminded that they could withdraw at any time without penalty. Informed verbal and written consent was obtained from parents prior to the administration of the Q-sort. The individual shown in Fig 1 of this manuscript has provided written informed consent (as outlined in PLOS consent form) to publish their image.

## Results

A total of 41 parents participated in the Q-sort task, and participant demographics regarding both parents and the children represented are presented in Table 2. The Q-sort took an average time of 13 minutes to complete. Two participants were excluded due to incomplete sorts. Three distinct viewpoints were defined by the remaining 39 participants. Six participants were mother and father dyads, representing three children with ABI. Of these three parental dyads recruited, only one pair had both parents aligned with the same viewpoint (viewpoint one), while one was split between viewpoint one and viewpoint two, and the other was split between viewpoints one and three. Distinguishing statements for each factor and the factor array or ‘typical sort’ of each statement for each factor are presented in Table 1. This table also presents seven consensus statements, and the seven most contended statements between viewpoints. Table 3 presents the individual participant demographics, arranged according factor loadings, or degree of alignment with their represented viewpoint. Statements defining each viewpoint are discussed. The statement’s relative positioning on the typical Q-sort for that viewpoint, i.e. (+5 strongly agree to -5 strongly disagree) will be included in parentheses as follows; (statement number, position).

**Table 2:** Participant Demographics.

	Factor 1	Factor 2	Factor 3
**Parents completing Q-sort**			
Mothers	13 (57%)	9 (100%)	4 (57%)
Fathers	10 (43%)	0	3 (43%)
Total	23	9	7
**Children represented**			
Total*	22	9	7
Age in years			
Mean	11.3	12.2	13.3
Median	12	13	14
Gender			
Female	8 (36%)	3 (33%)	2 (29%)
Male	14 (64%)	6 (67%)	5 (71%)
Time Since injury in years			
Mean	4.8	6.1	5.3
Range	0.25–13.4	1.1–15.4	0.25–13.7
Birthplace			
WA	14 (64%)	6 (67%)	1 (14%)
Australia—other	7 (32%)	1(11%)	3 (43%)
International	1 (4%)	2 (22%)	3 (43%)
Indigenous Australian			
Yes	3 (14%)	0	0
No	19 (86%)	9 (100%)	7 (100%)

*Two children whose parents load onto separate factors have been represented in both factors

**Table 3:** Individual participant demographics, arranged according to factor loading.

Parental Status	Age of child with ABI	Child Gender	Indigenous Status	Participant Factor Loading		
				Factor 1	Factor 2	Factor 3
Mother	6	Male	no	**0.789**	-0.001	0.255
Father	7	Male	no	**0.736**	-0.275	0.131
Father	12	Male	no	**0.726**	-0.084	-0.031
Mother	6	Female	no	**0.711**	0.078	0.152
Father	12	Female	yes	**0.709**	0.417	-0.114
Father	16	Male	no	**0.698**	-0.018	-0.093
mother	9	male	no	**0.695**	0.288	0.140
Mother	15	Male	no	**0.684**	-0.088	0.047
Mother	7	Male	yes	**0.670**	0.087	-0.258
Father	9	Female	no	**0.610**	0.313	-0.300
Mother	5	Female	no	**0.606**	0.204	0.083
Father*	13	Male	yes	**0.601**	0.134	-0.062
Mother	7	Male	yes	**0.582**	0.176	0.057
Mother*	13	Male	yes	**0.541**	0.346	-0.241
Father^	15	Male	no	**0.535**	0.275	-0.406
Mother	17	Male	no	**0.529**	0.367	0.219
Mother°	12	Male	no	**0.524**	0.299	0.135
Father	16	Female	no	**0.495**	-0.049	0.208
Mother	11	Male	no	**0.484**	0.125	0.134
Mother	10	Male	no	**0.471**	0.430	0.009
Mother	15	Female	no	**0.387**	-0.136	-0.482
Father	15	Female	no	**0.221**	-0.061	0.168
Father	13	Female	no	**0.065**	0.029	-0.366
Mother	8	Female	no	0.054	**0.686**	0.268
Mother	6	Female	no	0.158	**0.645**	0.061
Mother	17	Male	no	0.219	**0.585**	-0.080
Mother	13	Male	no	-0.065	**0.583**	-0.015
Mother	11	Female	no	-0.002	**0.459**	-0.155
Mother	10	Male	no	0.335	**0.446**	0.249
Mother	15	Male	no	0.312	**0.438**	0.154
Mother^	15	Male	no	0.049	**0.377**	0.237
Mother	15	Male	no	0.067	**0.302**	-0.070
Father°	12	Male	no	0.228	0.187	**0.521**
Mother	14	Male	no	0.183	0.428	**0.478**
Father	17	Female	no	0.407	0.194	**0.475**
Mother	11	Female	no	-0.323	0.350	**0.463**
Mother	17	Male	no	-0.020	-0.117	**0.414**
Father	14	Male	no	0.270	0.255	**0.369**
Mother	8	Male	no	0.103	-0.032	**0.351**

*^° Data from both mother and father collected, representing one child

### Viewpoint one: “We can participate!”

**Fig 3:**
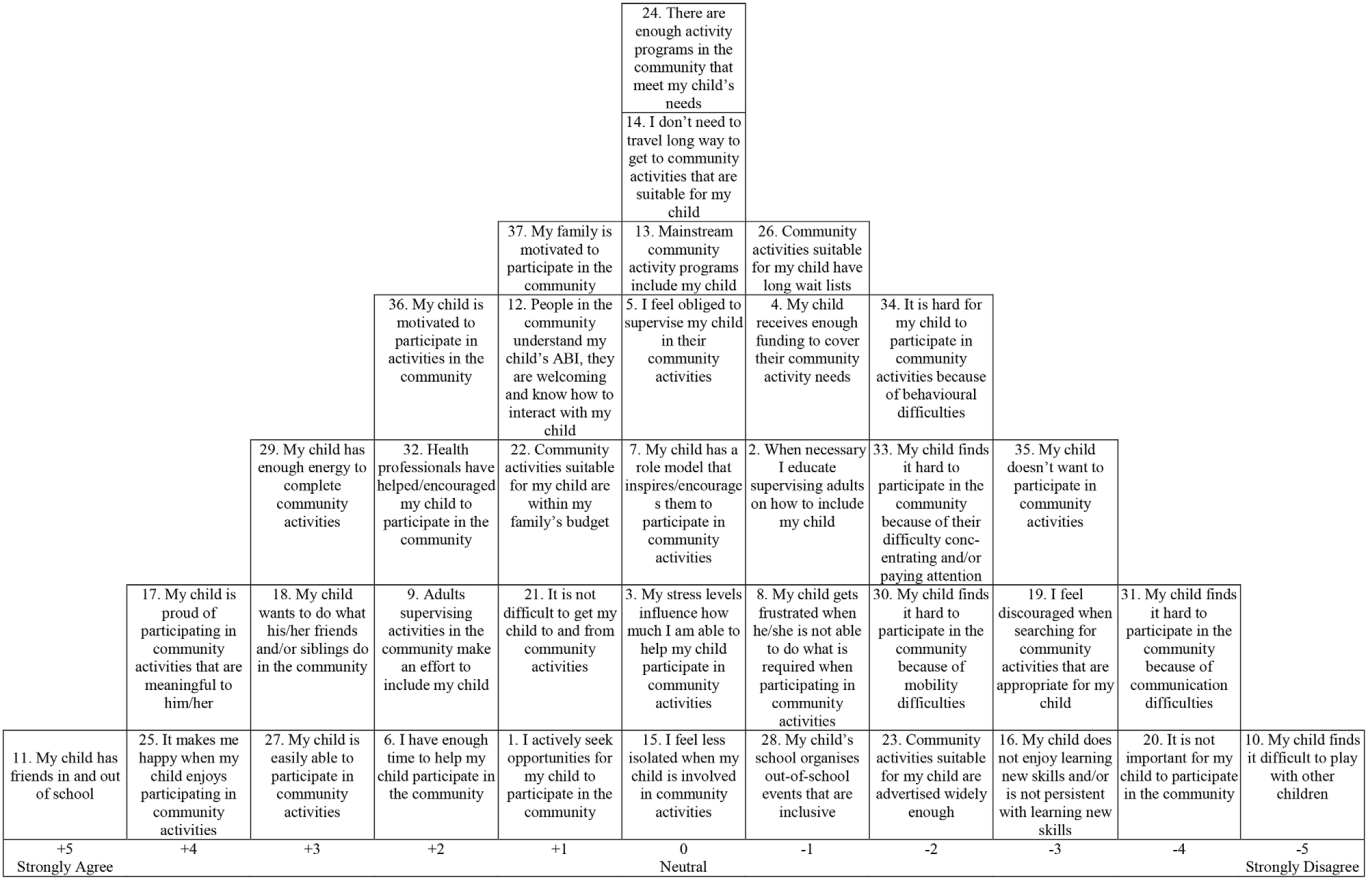
Typical Q Sort from Viewpoint One: We Can Participate.

Viewpoint one accounted for 26.8% of the variance, with 23 participants (56%) significantly associated with this factor. A total of 13 mothers and 10 fathers, (including one parental dyad), represented 22 children with ABI. Children were aged between 5 and 17 years old (with a mean of 11.3 years old) and were between 0.25–13.4 years post injury (a mean of 4.8 years post injury). This viewpoint included all three families from remote (Western) Australia, one family from outer regional (Western) Australia and all three of the Indigenous Australian children. Ten (44%) of the families in viewpoint one resided in areas of socio-economic disadvantage, eight families (35%) resided in advantaged areas and the remaining five families lived in average areas. The typical sort (Fig 3) demonstrates the average Q-sort arrangement of a parent from viewpoint one.

Viewpoint one was characterised by positive perspectives from parents regarding their child’s community participation; we *can* participate. The children were readily able to participate in community activities (statement 27; +3 agree) and were proud of doing so (17; +4). These children did not find it difficult to play (10; -5) with their friends, both in and out of school (11; +5), whom also motivated them to participate (18; +3). These children were able to focus their attention (33; -2), communicate (31; -4), manage their frustration (8; -1) and behave appropriately (34; -2) in community activities. Furthermore, these parents, relative to the other viewpoints, did not feel obliged to supervise their child (5; 0). Parents with viewpoint one, more so than the parents of other viewpoints, felt that people in the community understood, and were inclusive towards their child with an ABI (12; +1) and that health professionals had facilitated community participation through encouragement (32; +2).

Parents in viewpoint one felt they had enough time to facilitate community participation (6; +2), did not feel discouraged when searching for opportunities for their child (19; -3), and felt more positively than parents of other viewpoints about the availability of suitable activities (24; 0). Although these parents felt their child did not receive enough funding for community activities (4; -1), suitable activities were usually within their family’s budget (22; +1). Parents in viewpoint one felt that community participation was important (20; -4) and they strongly agreed that it made themselves feel happy when their child was engaged in community activities (25; +4).

### Viewpoint two: “We want to participate”

Viewpoint two accounted for 10.5% of the variance, with nine participants (23%) significantly associated with this factor. These participants were all mothers who identified as non-Indigenous. These nine parents represented children were aged between 6 and 17 years old (with a mean of 12.2 years old) and were between 1.1 and 15.4 years post injury (a mean of 6.1 years post injury). All of these families lived in major cities of Western Australia, with over half (56%) residing in areas of socio-economic advantage, two families (22%) residing in an area of socio-economic disadvantage and the remaining two (22%) living in socio-economically average areas.

The parents sharing viewpoint two felt very strongly that their children wanted to participate in community activities (we *want* to participate) (35; -5) and were proud of doing so (17; +4), even though they found participating somewhat difficult (27; -1). The children wanted to do what their friends/siblings did in the community (18; +4). However, they did not have friends both in and out of school (11; -1), found it more difficult to play with other children (10; +2) and experienced difficulties with communication (31; +1) and attention (33; +1).

Even though parents in viewpoint two strongly agreed that it made them happy when their child enjoyed participating (25; +5), family motivation to participate in the community was the lowest across all viewpoints (37; -1). These mothers indicated that their stress levels influenced how much they were able to facilitate their child’s community participation (3; +3), that they were not receiving enough funding (4; -4) and were experiencing financial constraints because of their family budget (22; -3). Compounding this, needing to travel long distances (14; -2) made getting their child to community activities difficult (21; -3). These mothers also felt that people in their communities were not always inclusive or understanding (12; -1) and that they had to educate supervising adults on how to include their child (2; +3).

### Viewpoint three: “We try to participate…”

Viewpoint three accounted for 7.6% of the variance with the remaining seven participants (18%) significantly associated with this factor. These participants represented seven non-indigenous children, all residing in metropolitan areas. Children were aged between 8 and 17 years old (mean of 13.3 years old) and ranged from 0.25–13.7 years (mean = 5.3 years) post injury. Of the seven families, one family (14%) resided in an area of socio-economic disadvantage, two (29%) resided in an average economic areas, and four (57%) resided in advantaged areas.

Parents in viewpoint three felt that their children found participating in the community difficult (27; +1), but were proud of participating in the community when they were able to (17; +5); we *try* to participate. Children represented in viewpoint three did have friends in and out of school (11; +2), and a role model that encouraged them to participate (7; +2). However, these children, as opposed to those in the other viewpoints, had mobility difficulties (30; +3), behavioural difficulties (34; +1) including managing frustration (8; +4), and experienced fatigue (29; -1), adversely impacting upon on their participation.

The parents felt happy when their child participated (25; +4) but felt the most obligated of the three viewpoints to supervise their children (5; +3). Parents in viewpoint three felt that child’s school did not organise inclusive out-of-school events (28; -4) and that community activities suitable for their children were not advertised widely enough (23; -4), leaving parents feeling discouraged when searching for opportunities (19; +2). Financially, suitable activities were not within the family’s budget (22; -1), nor within the child’s funding (4; -3). Parents in this viewpoint strongly felt that their child was not welcomed or included by people in their community (12; -5), nor into mainstream activities (13; -2).

### Consensus and contended statements

Consensus and contended statements are presented in Table 1 and are discussed below in the following format; (Statement number, Viewpoint one: position, Viewpoint two: position, Viewpoint three: position). Parents from all viewpoints strongly agreed that it made them happy when their child enjoyed participating (25; V1:+4, V2:+5, V3:+4) and agreed that they sought opportunities for their child to participate in the community (1; V1:+1, V2:+1, V3:+1). Parents also agreed that their children with ABI were motivated to participate (36; V1:+2, V2:+3, V3:0), were proud of participating (17; V1:+4, V2:+4, V3:+5), and enjoyed learning new skills (16; V1:-3, V2:-3, V3:-2). Suitable community activities did not have long wait lists (26; V1:-1, V2:-1, V3:-2), and parents agreed that feeling less isolated when their child participated in the community was not an important facilitator (15; V1:0, V2:+1, V3:0).

Contended statements (i.e. where the parent’s viewpoints differed the most) included whether or not children had friends both in and out of school (11; V1:+5, V2:-1, V3:+2), found it difficult to play with other children (10; V1:-5, V2:+2, V3:+3), or whether or not their mobility (30; V1:-2, V2:-2, V3:+3) or communication skills (31; V1:-4, V2:+1, V3:0), hindered their ability to participate in the community. Whether or not it was difficult to get their children to and from community activities (21; V1:+1, V2:-3, V3:-1) was also contended across the viewpoints. Parents in different viewpoints had different experiences with whether or not people in the community understood ABI and knew how to interact with and welcome their child (12; V1:+1, V2:-1, V3:-5), and whether or not their child’s school organised inclusive out-of-school events (28; V1:-1, V2:0, V3:-4).

In this study content validity was addressed by asking the participants for feedback regarding the relevancy of the statements [34]. Of the parents who responded to this question, most (n = 11/18) reported that the statements were relevant to their child and family, three stated they wanted more agree or disagree spaces on the grid and four parents felt the Q-sort was not relevant to their family. Face validity was assessed by asking parents how satisfied they were with their finalised sort on a scale of one ‘very unsatisfied’ to five ‘very satisfied’ [40]. The median satisfaction score reported was four, with a range from three to five. Only one suggestion for what could have been added to the Q-sort was made, which was to include continence issues.

## Discussion

While each of the three viewpoints revealed that parents agreed upon some of the facilitators and barriers to their child’s community participation each presented a unique perspective on this topic. The most significant consensus was that participating in meaningful activities made their child proud, and parents agreed that their child was motivated to participate and enjoyed learning new skills. It is likely that the experience of success contributed to positive participation experiences [23, 31], motivated continued participation [41, 42], and pointed to the importance of psychological factors in facilitating participation outcomes. Parents themselves were instrumental facilitators of their child’s participation, seeking opportunities for their children to participate and strongly agreeing that they felt happy when their child enjoyed community participation. Parents are instrumental in selecting the right opportunities for their child, maximising the psychological benefits of participation experiences [23].

Viewpoint one, “We can participate”, included the majority of the participants and suggested that these parents felt their children had good participation outcomes post-ABI. Due to the paucity of research into ABI in Australia [43], this is an important and encouraging finding. This viewpoint emphasised the child’s ability to participate, and the positive social support and attitudes that facilitated the family’s participation. Parents perceived their children as able, proud and motivated, with friends that facilitated their participation. Supportive relationships, including the child’s parents, health professionals, supervising adults and people in the community were all facilitators these children’s participation.

This viewpoint aligns strongly with literature reporting that supportive relationships facilitate children’s’ participation [12]. The presence of friendships and ease of play was a significant facilitator for the most prominent viewpoint in this study and the absence of these friendships was a significant barrier. Research into the lived experience of children with disabilities regarding their inclusion has also reported this dilemma; that while friendships facilitate participation, they are not always present [23]. A recent literature review described similar facilitators including experiencing a sense of connectedness, belonging and having authentic friendships, family support and role models to be essential elements contributing to meaningful participation experiences for children and youth with disabilities [31]. This suggests that supportive relationships should be a primary consideration when aiming to facilitate the community participation of children with ABI.

Of note to therapists, parents of adolescents with Down syndrome have reported that the social feature of group therapy interventions was a major facilitator of their child’s participation [44]. Parents of children with disabilities have emphasised the importance of opportunities for families in similar situations to network and support each other [23]. Building networks with other families of children with a disability supports family resilience [45], and participating as a family unit contributes to better participation outcomes for the child with a disability [12]. Embedding community based participation programs within a broader social framework such as a network of other programs or schools in which youth with disabilities and families are already connected contributes to better outcomes for children with disabilities [46]. These are all considerations for therapists and organisations aiming to facilitate better community participation for families of children with disabilities.

Viewpoint Two, “We want to participate…” demonstrated that families wanted to participate but were restricted by barriers at the environmental level. For these parents, participation was impeded by the parents’ stress levels, financial constraints and distance from community activities. The emphasis of the pragmatic aspects of community participation expressed by families within this viewpoint highlights the need for interventions which focus on remediating some of these environmental barriers.

Australian parents have suggested that modifying tasks and the environment would improve their child’s participation post ABI [43]. ‘Context therapy’ is an intervention that focuses solely on environmental and task modifications, and does not attempt to remediate impairments or factors specific to the child [47]. This approach has demonstrated effectiveness in studies with children with Cerebral palsy [47, 48]. A recent systematic review also concluded that context therapy is one of the most effective interventions for improving the participation outcomes of children with Cerebral palsy [49]. However, the appropriateness of this intervention in a participation setting with children with ABI is unknown. Given that many barriers to participation are environmental and contextual in nature for children with ABI, context therapy may prove to particularly effective in enhancing participation outcomes.

Family-centred practice has long been recognised as effective in improving the participation outcomes of children with disabilities [12, 50, 51]. However, while a family-centred approach to practice has been commended by parents, they have also called upon service providers to consider including practical strategies to minimise environmental barriers, such as the cost of programmes, inaccessible parking and organizing activities according to parental availability and geographical location [42]. While therapists are adept at grading and adapting activities and interventions to meet the specific needs of the children, working to empower and build parental capacity and address environmental barriers may also assist in improving participation outcomes for children with ABI.

As this viewpoint demonstrates, parents are key elements in the child’s environment, and have a major role in facilitating community participation outcomes. Occupational performance coaching aims to empower parents with the skills to create enabling environments for not only their child with a disability but their whole family [52]. This stepwise, structured approach is similar to that of solution-focused therapy and strengthens parent’s capability for problem-solving, and engenders a positive vision for the future [52]. More research is needed to understand the applicability of this approach to families living with a child with ABI.

Across the three viewpoints parents’ contested whether or not people in the community understood ABI, or whether they were welcoming and inclusive. The social model of disability, while recognising the impact of impairment, identifies that constraints in the social environment are often one of the most significant barriers to participation [53, 54]. According to children with disabilities, positive interpersonal interactions are central for inclusion [55]. Simply having the opportunity to participate is important for children with disabilities [31]. Findings from the present study show that when *communities* are inclusive of children with an ABI, they facilitate their participation in their community; however a lack of inclusive attitudes and actions are a clear barrier to community inclusion. Similarly, community program design has been found to be a major barrier to the inclusion of children with disabilities [23]. The community has an important role in social inclusion and future research should investigate public awareness of ABI and how this may affect their community participation [23, 56].

Viewpoint three, “We try to participate…” reflected that all aspects of the ICF: CY impacted upon their child’s ability to engage in meaningful community participation. Parents noted that participation was difficult. Environmental barriers such as societal attitudes and a lack of supportive relationships were emphasised, including being marginalised in the community and excluded from mainstream activities. This viewpoint highlighted that community participation is a multi-faceted experience, a finding also emphasised in other childhood participatory research [31]. These findings extend the ICF-CY definition of participation as involvement in a life situation, highlighting that *optimal* participation is a personal, subjective experience, through which children are able to derive meaning from their engagement in life [57]. While the ICF:CY has proved to be a useful model for use by researchers and therapists alike, this research adds to the growing call for the ICF-CY to encompass the important *subjective psychological aspects* of participation [31, 58, 59]. As occupational therapists have long since understood the link between participation and health [60], there is a clear opportunity to translate this paradigm into models of service delivery. This also supports the need to develop subjective measures of participation to capture the qualitative nature of engagement and meaning for children with disabilities [31, 59].

### Limitations

Due to the nature of data collection, no measure of impairment was administered, limiting the ability of the researchers to further analyse the relationship between the severity of ABI and the viewpoints discovered, beyond the moderate-severe’ ABI diagnosis. Participants were also purposively recruited from a hospital clinic and may not have been representative of all parents of children with ABI. This study addressed trustworthiness through the use of a steering group and by having expert researchers review findings to ensure confirmability [61]. Credibility was addressed by the primary researcher using reflective techniques throughout the data collection and analysis process [62]. Transferability was addressed by participants being chosen purposively (within a selection criteria), and description of demographic information and research context [62].

Although the pre-determined nature of Q methodology can be criticised [30], the statements were developed using a systematic review and were changed extensively following feedback from the steering group. The strength of Q-sort technique is it forces prioritisation of items to reveal viewpoints, a strength which was key in choosing this methodology [30]. While the small sample size was appropriate for discovering the finite set of viewpoints as in Q-sort methodology literature (35), a Q sort study can never be completely exhaustive, but provides a distillation of available information [39]. Overall, this research demonstrated that Q-methodology is a robust research method that can be used in busy clinical environments.

## Conclusions

This study demonstrates that community participation is a complex experience and service providers and community recreation organisations targeting this outcome must take into consideration environmental barriers. Three viewpoints were identified regarding barriers and facilitators to community participation in children with ABI; Viewpoint one: “We can participate!”, Viewpoint two: “We want to participate…” and Viewpoint three: “We try to participate…”. These findings indicate good participation outcomes for most children and families, however some families who were motivated to participate experienced significant barriers. The most significant facilitators included motivation and supportive relationships from immediate family, friends and community attitudes. The lack of supportive relationships and attitudes was perceived as a fundamental barrier to community participation.

Q-methodology proved effective in not only uncovering prominent viewpoints from parents but also in identifying significant barriers and facilitators impacting the community participation of children with ABI. The successful implementation of Q-sorts in a busy clinical environment should encourage further use of this methodology by therapists and service providers.

**Key Points**

- The most prominent viewpoint of parents who have children with Acquired Brain Injury in Western Australia was that their family is able to participate in the community, due to supportive relationships and attitudes.
- Children having friends, feeling proud of participating and parents being pleased as a result are significant facilitators for participation.
- A lack of friends and unsupportive or exclusive community attitudes were barriers to community participation for children with an ABI.
- Future research should examine the effectiveness of interventions at the environmental level (family-centred practice, context therapy and occupational performance coaching) in removing barriers to community participation for children with ABI.

## Supporting Information

S1 File Minimal Dataset. (DOCX)
