# Supplementary material for: Can, Want and Try: Parents’ Viewpoints Regarding the Participation of Their Child with an Acquired Brain Injury
Source: PLoS One. 2016 Jul 1;11(7):e0157951. doi: 10.1371/journal.pone.0157951 (PMC4930177; doi:10.1371/journal.pone.0157951)
Supplement: S1 File — (DOCX) [file pone.0157951.s001.docx]

S1 File. Minimal Dataset.

PQMethod2.35 ABIandcommunityparticipation PAGE 1

Path and Project Name: C:\PQMETHOD\projects/abione 2/14 20

Correlation Matrix Between Sorts

SORTS 1 2 3 4 5 6 7 8 9 10 11 12 13 14 15 16 17 18 19 20 21 22 23 24 25 26 27 28 29 30

1 101 100 35 16 18 42 33 15 41 36 47 -10 43 30 40 27 49 42 49 48 27 59 20 50 38 -11 42 41 61 35 49

2 103 35 100 7 29 0 11 36 45 18 27 32 19 22 22 9 5 20 0 -2 4 31 -16 33 24 -13 26 31 22 46 20

3 104 16 7 100 20 3 -18 6 26 14 28 10 1 -5 -10 2 10 -11 -11 11 5 21 0 3 20 10 41 1 -11 13 50

4 202 18 29 20 100 29 30 10 41 3 25 27 52 32 23 -27 0 18 35 35 1 22 20 28 24 -12 18 24 12 23 30

5 201 42 0 3 29 100 36 9 38 51 43 -37 50 11 33 18 36 43 62 58 19 54 34 24 37 -4 6 36 49 -1 26

6 301 33 11 -18 30 36 100 0 22 34 44 -39 61 -2 36 16 30 32 32 17 17 42 39 18 38 -42 -22 28 31 10 16

7 302 15 36 6 10 9 0 100 3 9 12 18 -3 26 32 -5 -5 2 24 8 20 5 -19 12 23 15 26 20 -12 31 12

8 303 41 45 26 41 38 22 3 100 31 55 3 52 5 38 9 37 32 41 22 28 43 5 33 26 -7 32 47 48 20 40

9 304 36 18 14 3 51 34 9 31 100 55 -17 39 -17 24 8 47 56 35 36 40 71 20 6 38 -10 14 49 45 13 22

10 305 47 27 28 25 43 44 12 55 55 100 -17 60 -19 41 20 30 44 30 30 27 60 32 17 54 -16 6 60 51 23 45

11 401 -10 32 10 27 -37 -39 18 3 -17 -17 100 -22 30 -7 -20 -38 -10 -20 -8 -19 -5 -41 12 -18 20 21 1 -23 12 25

12 402 43 19 1 52 50 61 -3 52 39 60 -22 100 -5 30 0 24 53 43 30 17 47 50 18 34 -33 -6 43 43 18 23

13 501 30 22 -5 32 11 -2 26 5 -17 -19 30 -5 100 16 -16 -12 -13 35 26 -4 -3 -10 50 -18 -3 32 12 2 28 -1

14 502 40 22 -10 23 33 36 32 38 24 41 -7 30 16 100 18 37 24 47 38 11 33 -3 35 39 1 16 46 41 11 30

15 601 27 9 2 -27 18 16 -5 9 8 20 -20 0 -16 18 100 24 12 -4 3 16 11 -14 -10 20 24 17 7 18 12 35

16 602 49 5 10 0 36 30 -5 37 47 30 -38 24 -12 37 24 100 52 34 45 66 52 17 38 40 21 18 11 48 17 23

17 701 42 20 -11 18 43 32 2 32 56 44 -10 53 -13 24 12 52 100 34 47 52 55 41 20 46 5 13 41 63 22 26

18 702 49 0 -11 35 62 32 24 41 35 30 -20 43 35 47 -4 34 34 100 60 18 32 20 35 22 -12 15 40 46 19 11

19 703 48 -2 11 35 58 17 8 22 36 30 -8 30 26 38 3 45 47 60 100 24 53 13 28 42 6 33 31 48 17 25

20 704 27 4 5 1 19 17 20 28 40 27 -19 17 -4 11 16 66 52 18 24 100 32 20 22 28 27 26 28 18 33 10

21 801 59 31 21 22 54 42 5 43 71 60 -5 47 -3 33 11 52 55 32 53 32 100 11 33 48 2 23 52 55 33 52

22 802 20 -16 0 20 34 39 -19 5 20 32 -41 50 -10 -3 -14 17 41 20 13 20 11 100 -6 29 -35 -10 2 27 -11 -16

23 803 50 33 3 28 24 18 12 33 6 17 12 18 50 35 -10 38 20 35 28 22 33 -6 100 29 -2 5 27 18 46 28

24 804 38 24 20 24 37 38 23 26 38 54 -18 34 -18 39 20 40 46 22 42 28 48 29 29 100 -8 2 36 30 31 29

25 901 -11 -13 10 -12 -4 -42 15 -7 -10 -16 20 -33 -3 1 24 21 5 -12 6 27 2 -35 -2 -8 100 36 -5 -7 9 26

26 902 42 26 41 18 6 -22 26 32 14 6 21 -6 32 16 17 18 13 15 33 26 23 -10 5 2 36 100 20 18 25 45

27 903 41 31 1 24 36 28 20 47 49 60 1 43 12 46 7 11 41 40 31 28 52 2 27 36 -5 20 100 33 32 34

28 904 61 22 -11 12 49 31 -12 48 45 51 -23 43 2 41 18 48 63 46 48 18 55 27 18 30 -7 18 33 100 -4 23

29 905 35 46 13 23 -1 10 31 20 13 23 12 18 28 11 12 17 22 19 17 33 33 -11 46 31 9 25 32 -4 100 29

30 906 49 20 50 30 26 16 12 40 22 45 25 23 -1 30 35 23 26 11 25 10 52 -16 28 29 26 45 34 23 29 100

31 907 26 44 6 32 -8 16 37 28 -5 22 22 18 39 44 -12 5 -10 18 17 1 20 -27 50 20 -15 18 41 -9 37 20

32 908 49 43 4 23 15 37 25 25 9 31 10 8 32 32 26 25 6 17 22 12 38 -5 39 38 10 30 28 27 27 46

33 1001 15 -18 -14 -1 19 32 4 -17 -7 -9 -9 18 11 2 28 2 14 20 17 4 -8 15 -18 -10 -7 -8 -5 8 -14 -1

34 1002 57 7 13 40 49 51 3 33 43 51 -18 53 28 54 16 48 45 44 63 29 59 29 43 59 -4 21 48 49 23 39

35 1003 -4 41 -15 13 -9 18 7 30 -25 -19 -8 15 11 11 23 3 -6 -1 -31 0 -28 8 -3 -5 -16 9 -18 0 3 -16

36 1004 30 13 10 35 23 24 1 54 42 43 -2 26 -3 31 -5 46 32 37 26 39 45 21 28 30 0 31 22 39 13 24

37 1005 9 24 -7 45 17 30 36 44 0 21 21 31 19 60 -9 10 10 22 13 9 23 -14 31 27 6 6 42 12 7 32

38 1101 46 19 -12 10 50 25 21 43 57 39 0 37 29 43 10 50 43 54 48 40 58 9 29 22 10 29 39 43 29 21

39 1102 3 9 -13 28 1 24 14 10 -21 -8 11 24 25 18 18 -5 20 7 2 12 -21 15 13 8 9 13 10 5 -8 8

PQMethod2.35 ABIandcommunityparticipation PAGE 2

Path and Project Name: C:\PQMETHOD\projects/abione 2/14 20

Correlation Matrix Between Sorts

SORTS 31 32 33 34 35 36 37 38 39

1 101 26 49 15 57 -4 30 9 46 3

2 103 44 43 -18 7 41 13 24 19 9

3 104 6 4 -14 13 -15 10 -7 -12 -13

4 202 32 23 -1 40 13 35 45 10 28

5 201 -8 15 19 49 -9 23 17 50 1

6 301 16 37 32 51 18 24 30 25 24

7 302 37 25 4 3 7 1 36 21 14

8 303 28 25 -17 33 30 54 44 43 10

9 304 -5 9 -7 43 -25 42 0 57 -21

10 305 22 31 -9 51 -19 43 21 39 -8

11 401 22 10 -9 -18 -8 -2 21 0 11

12 402 18 8 18 53 15 26 31 37 24

13 501 39 32 11 28 11 -3 19 29 25

14 502 44 32 2 54 11 31 60 43 18

15 601 -12 26 28 16 23 -5 -9 10 18

16 602 5 25 2 48 3 46 10 50 -5

17 701 -10 6 14 45 -6 32 10 43 20

18 702 18 17 20 44 -1 37 22 54 7

19 703 17 22 17 63 -31 26 13 48 2

20 704 1 12 4 29 0 39 9 40 12

21 801 20 38 -8 59 -28 45 23 58 -21

22 802 -27 -5 15 29 8 21 -14 9 15

23 803 50 39 -18 43 -3 28 31 29 13

24 804 20 38 -10 59 -5 30 27 22 8

25 901 -15 10 -7 -4 -16 0 6 10 9

26 902 18 30 -8 21 9 31 6 29 13

27 903 41 28 -5 48 -18 22 42 39 10

28 904 -9 27 8 49 0 39 12 43 5

29 905 37 27 -14 23 3 13 7 29 -8

30 906 20 46 -1 39 -16 24 32 21 8

31 907 100 50 -30 40 7 9 52 33 21

32 908 50 100 -9 50 7 14 30 35 27

33 1001 -30 -9 100 4 24 -22 4 -10 18

34 1002 40 50 4 100 -10 36 42 54 28

35 1003 7 7 24 -10 100 9 24 -5 39

36 1004 9 14 -22 36 9 100 28 31 -12

37 1005 52 30 4 42 24 28 100 25 33

38 1101 33 35 -10 54 -5 31 25 100 0

39 1102 21 27 18 28 39 -12 33 0 100

PQMethod2.35 ABIandcommunityparticipation PAGE 3

Path and Project Name: C:\PQMETHOD\projects/abione 2/14 20

Unrotated Factor Matrix

Factors

1 2 3 4 5 6 7

SORTS

1 101 0.7578 -0.0113 0.1022 -0.0667 0.0094 0.1501 -0.1262

2 103 0.3969 0.4915 0.2134 -0.2403 0.1390 -0.1452 -0.1030

3 104 0.1047 0.1236 0.3291 -0.1538 0.0664 0.1100 0.3376

4 202 0.4567 0.2865 -0.3258 -0.0071 0.0581 0.1090 0.4789

5 201 0.6114 -0.3620 -0.1314 0.0584 0.0521 0.0906 0.1275

6 301 0.5669 -0.2501 -0.3782 -0.2924 0.0997 -0.3093 -0.1752

7 302 0.2356 0.3801 0.0131 0.1982 0.0658 -0.1373 -0.1244

8 303 0.6541 0.1596 0.0955 -0.1335 0.0230 0.1094 0.2248

9 304 0.5344 -0.4406 0.3929 0.1708 0.1574 -0.2508 0.1641

10 305 0.6769 -0.1633 0.2233 -0.2507 0.0616 -0.3142 0.2704

11 401 -0.0873 0.6424 0.1442 0.0562 -0.1646 0.0600 0.2215

12 402 0.6593 -0.1969 -0.2945 -0.2744 0.0677 -0.0640 0.2326

13 501 0.2098 0.4410 -0.3253 0.3379 0.1423 0.2346 -0.1260

14 502 0.6152 0.1148 -0.1234 0.1393 0.0119 -0.1235 -0.0292

15 601 0.1809 -0.0468 0.2144 -0.3035 0.0617 0.0687 -0.3976

16 602 0.5684 -0.3295 0.2160 0.1477 0.0724 0.1884 -0.2800

17 701 0.6010 -0.3865 0.1566 0.0076 0.0707 0.0991 0.0099

18 702 0.6178 -0.1112 -0.2756 0.3301 0.0613 0.1660 0.0434

19 703 0.5877 -0.1926 -0.0059 0.3571 0.0580 0.1998 0.1717

20 704 0.4315 -0.1327 0.2940 0.2015 0.0618 0.1407 -0.2269

21 801 0.7175 -0.1975 0.3643 0.0853 0.0785 -0.1862 0.1694

22 802 0.2411 -0.5253 -0.2576 -0.2389 0.1469 0.0501 0.1151

23 803 0.4898 0.2680 -0.0376 0.2250 0.0416 0.0024 -0.1197

24 804 0.6001 -0.0892 0.0690 -0.0615 0.0090 -0.2074 0.0156

25 901 -0.0384 0.1491 0.4022 0.2646 -0.0841 0.3156 -0.0962

26 902 0.3300 0.3218 0.3827 0.0144 0.1058 0.4775 0.0108

27 903 0.6114 0.0662 0.0594 0.1066 0.0078 -0.2959 0.1686

28 904 0.6018 -0.3608 0.0657 -0.0326 0.0534 0.1253 0.0320

29 905 0.3857 0.2701 0.2285 0.0633 0.0539 -0.0674 -0.1882

30 906 0.4875 0.2253 0.3750 -0.1672 0.0954 0.0985 0.1683

31 907 0.3848 0.6186 -0.1189 0.1582 0.1538 -0.4446 -0.0617

32 908 0.5212 0.3149 0.0424 -0.0956 0.0418 -0.0528 -0.2607

33 1001 0.0419 -0.2056 -0.3081 -0.0880 0.0445 0.2661 -0.1611

34 1002 0.8096 -0.0622 -0.1739 0.0749 0.0084 -0.0111 0.0290

35 1003 0.0737 0.2087 -0.2269 -0.4027 0.0916 0.1458 -0.3868

36 1004 0.4961 -0.0424 0.1401 0.0634 0.0134 0.0896 0.2528

37 1005 0.4417 0.4322 -0.2512 0.0813 0.0854 -0.1276 0.0952

38 1101 0.6367 -0.0655 0.0807 0.4654 0.0835 -0.0463 -0.0602

39 1102 0.1972 0.2479 -0.3666 -0.1330 0.0675 0.2136 -0.1302

Eigenvalues 9.7422 3.5143 2.2828 1.6151 0.2679 1.4692 1.5230

% expl.Var. 25 9 6 4 1 4 4

PQMethod2.35 ABIandcommunityparticipation PAGE 4

Path and Project Name: C:\PQMETHOD\projects/abione 2/14 20

Cumulative Communalities Matrix

Factors 1 Thru ....

1 2 3 4 5 6 7

SORTS

1 101 0.5743 0.5744 0.5849 0.5894 0.5894 0.6120 0.6279

2 103 0.1576 0.3991 0.4446 0.5024 0.5217 0.5428 0.5534

3 104 0.0110 0.0262 0.1346 0.1582 0.1626 0.1747 0.2887

4 202 0.2086 0.2907 0.3968 0.3969 0.4002 0.4121 0.6415

5 201 0.3738 0.5049 0.5222 0.5256 0.5283 0.5365 0.5528

6 301 0.3214 0.3839 0.5269 0.6124 0.6224 0.7180 0.7487

7 302 0.0555 0.2000 0.2002 0.2395 0.2438 0.2626 0.2781

8 303 0.4278 0.4533 0.4624 0.4803 0.4808 0.4928 0.5433

9 304 0.2856 0.4798 0.6341 0.6633 0.6881 0.7510 0.7779

10 305 0.4581 0.4848 0.5346 0.5975 0.6013 0.7000 0.7731

11 401 0.0076 0.4203 0.4411 0.4443 0.4714 0.4750 0.5240

12 402 0.4347 0.4735 0.5602 0.6356 0.6401 0.6442 0.6983

13 501 0.0440 0.2385 0.3444 0.4585 0.4788 0.5338 0.5497

14 502 0.3784 0.3916 0.4069 0.4262 0.4264 0.4417 0.4425

15 601 0.0327 0.0349 0.0809 0.1730 0.1768 0.1815 0.3396

16 602 0.3230 0.4316 0.4782 0.5000 0.5053 0.5408 0.6192

17 701 0.3612 0.5106 0.5351 0.5351 0.5401 0.5500 0.5501

18 702 0.3817 0.3940 0.4700 0.5790 0.5827 0.6103 0.6121

19 703 0.3454 0.3825 0.3825 0.5100 0.5134 0.5533 0.5828

20 704 0.1861 0.2038 0.2902 0.3308 0.3346 0.3544 0.4059

21 801 0.5149 0.5539 0.6866 0.6939 0.7000 0.7347 0.7634

22 802 0.0581 0.3341 0.4004 0.4575 0.4791 0.4816 0.4948

23 803 0.2399 0.3117 0.3131 0.3637 0.3655 0.3655 0.3798

24 804 0.3601 0.3681 0.3729 0.3766 0.3767 0.4197 0.4200

25 901 0.0015 0.0237 0.1855 0.2555 0.2625 0.3621 0.3714

26 902 0.1089 0.2124 0.3589 0.3591 0.3703 0.5983 0.5985

27 903 0.3738 0.3782 0.3817 0.3931 0.3931 0.4807 0.5091

28 904 0.3622 0.4924 0.4967 0.4978 0.5006 0.5163 0.5173

29 905 0.1487 0.2217 0.2739 0.2779 0.2808 0.2853 0.3208

30 906 0.2376 0.2884 0.4290 0.4570 0.4661 0.4758 0.5041

31 907 0.1481 0.5307 0.5448 0.5699 0.5935 0.7911 0.7949

32 908 0.2717 0.3709 0.3727 0.3818 0.3836 0.3863 0.4543

33 1001 0.0018 0.0440 0.1389 0.1467 0.1487 0.2195 0.2454

34 1002 0.6555 0.6594 0.6896 0.6952 0.6953 0.6954 0.6962

35 1003 0.0054 0.0490 0.1005 0.2626 0.2710 0.2923 0.4419

36 1004 0.2461 0.2479 0.2675 0.2715 0.2717 0.2797 0.3437

37 1005 0.1951 0.3819 0.4450 0.4516 0.4589 0.4752 0.4843

38 1101 0.4053 0.4096 0.4161 0.6327 0.6397 0.6418 0.6454

39 1102 0.0389 0.1003 0.2347 0.2524 0.2570 0.3026 0.3195

cum% expl.Var. 25 34 40 44 45 48 52

PQMethod2.35 ABIandcommunityparticipation PAGE 5

Path and Project Name: C:\PQMETHOD\projects/abione 2/14 20

Factor Matrix with an X Indicating a Defining Sort

Loadings

QSORT 1 2 3

1 101 0.6946X 0.2878 0.1402

2 103 0.1828 0.4279 0.4776X

3 104 0.1032 -0.0321 0.3505

4 202 0.2193 0.5852X -0.0795

5 201 0.6695X 0.0872 -0.2576

6 301 0.5351X 0.2749 -0.4062

7 302 0.0490 0.3765 0.2367

8 303 0.5288X 0.3671 0.2192

9 304 0.7356X -0.2753 0.1314

10 305 0.7109X 0.0782 0.1521

11 401 -0.3229 0.3501 0.4629X

12 402 0.6103X 0.3128 -0.3000

13 501 -0.0646 0.5831X -0.0148

14 502 0.4714X 0.4296 0.0089

15 601 0.2210 -0.0611 0.1682

16 602 0.6843X -0.0881 0.0469

17 701 0.7260X -0.0837 -0.0311

18 702 0.5407X 0.3458 -0.2409

19 703 0.6005X 0.1341 -0.0624

20 704 0.4945X -0.0490 0.2080

21 801 0.7885X -0.0005 0.2546

22 802 0.3873 -0.1361 -0.4815

23 803 0.3118 0.4384X 0.1538

24 804 0.5819X 0.1760 0.0574

25 901 -0.0201 -0.1172 0.4139

26 902 0.2283 0.1872 0.5213X

27 903 0.5239X 0.2985 0.1348

28 904 0.6984X -0.0179 -0.0930

29 905 0.2699 0.2549 0.3688

30 906 0.4072 0.1939 0.4750X

31 907 0.0541 0.6855X 0.2683

32 908 0.3350 0.4456X 0.2487

33 1001 0.0654 0.0294 -0.3658

34 1002 0.7090X 0.4171 -0.1139

35 1003 -0.0673 0.3017 -0.0700

36 1004 0.4836X 0.1253 0.1339

37 1005 0.1583 0.6451X 0.0609

38 1101 0.6063X 0.2039 0.0831

39 1102 -0.0016 0.4588X -0.1554

% expl.Var. 23 10 7

PQMethod2.35 ABIandcommunityparticipation PAGE 6

Path and Project Name: C:\PQMETHOD\projects/abione 2/14 20

Free Distribution Data Results

QSORT MEAN ST.DEV.

1 101 0.000 2.415

2 103 0.000 2.415

3 104 0.000 2.415

4 202 0.000 2.415

5 201 0.000 2.415

6 301 0.000 2.415

7 302 0.000 2.415

8 303 0.000 2.415

9 304 0.000 2.415

10 305 0.000 2.415

11 401 0.000 2.415

12 402 0.000 2.415

13 501 0.000 2.415

14 502 0.000 2.415

15 601 0.000 2.415

16 602 0.000 2.415

17 701 0.000 2.415

18 702 0.000 2.415

19 703 0.000 2.415

20 704 0.000 2.415

21 801 0.000 2.415

22 802 0.000 2.415

23 803 0.000 2.415

24 804 0.000 2.415

25 901 0.000 2.415

26 902 0.000 2.415

27 903 0.000 2.415

28 904 0.000 2.415

29 905 0.000 2.415

30 906 0.000 2.415

31 907 0.000 2.415

32 908 0.000 2.415

33 1001 0.000 2.415

34 1002 0.000 2.415

35 1003 0.000 2.415

36 1004 0.000 2.415

37 1005 0.000 2.415

38 1101 0.000 2.415

39 1102 0.000 2.415

PQMethod2.35 ABIandcommunityparticipation PAGE 7

Path and Project Name: C:\PQMETHOD\projects/abione 2/14 20

Factor Scores with Corresponding Ranks

Factors

No. Statement No. 1 2 3

1 I actively seek opportunities for my child to particip 1 0.66 12 0.44 14 0.47 11

2 When necessary I educate supervising adults on how to 2 -0.20 23 1.04 5 0.39 14

3 My stress levels influence how much I am able to help 3 0.03 20 1.29 4 0.40 13

4 My child receives enough funding to cover their commun 4 -0.57 25 -1.42 35 -1.10 33

5 I feel obliged to supervise my child in their communit 5 -0.14 22 0.68 9 0.94 6

6 I have enough time to help my child participate in the 6 0.73 10 -0.89 30 -0.57 27

7 My child has a role model that inspires/encourages the 7 0.32 17 -0.02 18 0.79 8

8 My child gets frustrated when he/she is not able to do 8 -0.49 24 0.02 17 1.20 3

9 Adults supervising activities in the cmmty make an eff 9 0.77 9 0.88 7 -0.05 22

10 My child finds it difficult to play with other childre 10 -1.86 37 0.57 10 0.47 12

11 My child has friends in and out of school 11 2.04 1 -0.31 24 0.87 7

12 People in the community understand my child’s ABI, the 12 0.51 15 -0.37 25 -2.61 37

13 Mainstream community activity programs include my chil 13 0.37 16 0.21 16 -0.60 28

14 I don’t need to travel long way to get to community ac 14 0.26 18 -0.58 28 0.29 16

15 I feel less isolated when my child is involved in comm 15 0.19 19 0.52 12 0.11 21

16 My child does not enjoy learning new skills and/or is 16 -1.27 34 -1.17 32 -0.67 30

17 My child is proud of participating in community activi 17 1.22 3 1.69 3 2.00 1

18 My child wants to do what his/her friends and/or sibli 18 0.98 5 1.96 2 1.12 5

19 I feel discouraged when searching for community activi 19 -1.21 32 0.52 13 0.49 10

20 It is not important for my child to participate in the 20 -1.77 36 -1.44 36 -0.76 31

21 It is not difficult to get my child to and from commun 21 0.54 14 -1.40 34 0.64 9

22 Community activities suitable for my child are within 22 0.54 13 -1.27 33 -0.12 24

23 Community activities suitable for my child are adverti 23 -0.62 28 -0.21 21 -1.81 35

24 There are enough activity programs in the community th 24 -0.10 21 -0.68 29 -1.27 34

25 It makes me happy when my child enjoys participating 25 1.90 2 2.29 1 1.71 2

26 Community activities suitable for my child have long w 26 -0.59 26 -0.26 23 -0.64 29

27 My child is easily able to participate in community ac 27 1.15 4 -0.46 26 -0.14 25

28 My child’s school organises out-of-school events that 28 -0.60 27 -0.16 20 -2.28 36

29 My child has enough energy to complete community activ 29 0.96 6 0.78 8 -0.40 26

30 My child finds it hard to participate in the cmmty bo 30 -1.00 29 -1.12 31 1.12 4

31 My child finds it hard to participate in the cmmty bo 31 -1.60 35 0.35 15 0.14 20

32 Health professionals have helped/encouraged my child t 32 0.77 8 -0.25 22 -0.11 23

33 My child finds it hard to participate in cmmty bo diff 33 -1.07 30 0.56 11 0.14 19

34 It is hard for my child to participate in cmmty act bo 34 -1.13 31 -0.10 19 0.30 15

35 My child doesn’t want to participate in community acti 35 -1.26 33 -2.06 37 -0.83 32

36 My child is motivated to participate in activities in 36 0.88 7 0.92 6 0.24 17

37 My family is motivated to participate in the community 37 0.66 11 -0.55 27 0.15 18

PQMethod2.35 ABIandcommunityparticipation PAGE 8

Path and Project Name: C:\PQMETHOD\projects/abione 2/14 20

Correlations Between Factor Scores

1 2 3

1 1.0000 0.3583 0.2767

2 0.3583 1.0000 0.4901

3 0.2767 0.4901 1.0000

PQMethod2.35 ABIandcommunityparticipation PAGE 9

Path and Project Name: C:\PQMETHOD\projects/abione 2/14 20

Factor Scores -- For Factor 1

No. Statement No. Z-SCORES

11 My child has friends in and out of school 11 2.039

25 It makes me happy when my child enjoys participating in com 25 1.904

17 My child is proud of participating in community activities t 17 1.221

27 My child is easily able to participate in community activiti 27 1.148

18 My child wants to do what his/her friends and/or siblings do 18 0.980

29 My child has enough energy to complete community activities 29 0.964

36 My child is motivated to participate in activities in the co 36 0.884

32 Health professionals have helped/encouraged my child to part 32 0.771

9 Adults supervising activities in the cmmty make an effrt to 9 0.770

6 I have enough time to help my child participate in the commu 6 0.731

37 My family is motivated to participate in the community 37 0.660

1 I actively seek opportunities for my child to participate in 1 0.656

22 Community activities suitable for my child are within my fa 22 0.539

21 It is not difficult to get my child to and from community ac 21 0.537

12 People in the community understand my child’s ABI, they are 12 0.507

13 Mainstream community activity programs include my child 13 0.367

7 My child has a role model that inspires/encourages them to p 7 0.317

14 I don’t need to travel long way to get to community activiti 14 0.261

15 I feel less isolated when my child is involved in community 15 0.186

3 My stress levels influence how much I am able to help my chi 3 0.032

24 There are enough activity programs in the community that mee 24 -0.101

5 I feel obliged to supervise my child in their community acti 5 -0.140

2 When necessary I educate supervising adults on how to includ 2 -0.201

8 My child gets frustrated when he/she is not able to do what 8 -0.485

4 My child receives enough funding to cover their community ac 4 -0.572

26 Community activities suitable for my child have long wait li 26 -0.588

28 My child’s school organises out-of-school events that are in 28 -0.599

23 Community activities suitable for my child are advertised wi 23 -0.615

30 My child finds it hard to participate in the cmmty bo mobili 30 -1.003

33 My child finds it hard to participate in cmmty bo difficulty 33 -1.069

34 It is hard for my child to participate in cmmty act bo behav 34 -1.125

19 I feel discouraged when searching for community activities t 19 -1.215

35 My child doesn’t want to participate in community activities 35 -1.256

16 My child does not enjoy learning new skills and/or is not pe 16 -1.270

31 My child finds it hard to participate in the cmmty bo commun 31 -1.601

20 It is not important for my child to participate in the commu 20 -1.773

10 My child finds it difficult to play with other children 10 -1.863

PQMethod2.35 ABIandcommunityparticipation PAGE 10

Path and Project Name: C:\PQMETHOD\projects/abione 2/14 20

Factor Scores -- For Factor 2

No. Statement No. Z-SCORES

25 It makes me happy when my child enjoys participating in com 25 2.292

18 My child wants to do what his/her friends and/or siblings do 18 1.956

17 My child is proud of participating in community activities t 17 1.693

3 My stress levels influence how much I am able to help my chi 3 1.287

2 When necessary I educate supervising adults on how to includ 2 1.043

36 My child is motivated to participate in activities in the co 36 0.921

9 Adults supervising activities in the cmmty make an effrt to 9 0.883

29 My child has enough energy to complete community activities 29 0.780

5 I feel obliged to supervise my child in their community acti 5 0.679

10 My child finds it difficult to play with other children 10 0.571

33 My child finds it hard to participate in cmmty bo difficulty 33 0.564

15 I feel less isolated when my child is involved in community 15 0.520

19 I feel discouraged when searching for community activities t 19 0.515

1 I actively seek opportunities for my child to participate in 1 0.443

31 My child finds it hard to participate in the cmmty bo commun 31 0.346

13 Mainstream community activity programs include my child 13 0.212

8 My child gets frustrated when he/she is not able to do what 8 0.024

7 My child has a role model that inspires/encourages them to p 7 -0.018

34 It is hard for my child to participate in cmmty act bo behav 34 -0.103

28 My child’s school organises out-of-school events that are in 28 -0.161

23 Community activities suitable for my child are advertised wi 23 -0.210

32 Health professionals have helped/encouraged my child to part 32 -0.253

26 Community activities suitable for my child have long wait li 26 -0.264

11 My child has friends in and out of school 11 -0.310

12 People in the community understand my child’s ABI, they are 12 -0.369

27 My child is easily able to participate in community activiti 27 -0.465

37 My family is motivated to participate in the community 37 -0.555

14 I don’t need to travel long way to get to community activiti 14 -0.576

24 There are enough activity programs in the community that mee 24 -0.679

6 I have enough time to help my child participate in the commu 6 -0.890

30 My child finds it hard to participate in the cmmty bo mobili 30 -1.119

16 My child does not enjoy learning new skills and/or is not pe 16 -1.168

22 Community activities suitable for my child are within my fa 22 -1.269

21 It is not difficult to get my child to and from community ac 21 -1.404

4 My child receives enough funding to cover their community ac 4 -1.421

20 It is not important for my child to participate in the commu 20 -1.440

35 My child doesn’t want to participate in community activities 35 -2.056

PQMethod2.35 ABIandcommunityparticipation PAGE 11

Path and Project Name: C:\PQMETHOD\projects/abione 2/14 20

Factor Scores -- For Factor 3

No. Statement No. Z-SCORES

17 My child is proud of participating in community activities t 17 2.002

25 It makes me happy when my child enjoys participating in com 25 1.709

8 My child gets frustrated when he/she is not able to do what 8 1.200

30 My child finds it hard to participate in the cmmty bo mobili 30 1.123

18 My child wants to do what his/her friends and/or siblings do 18 1.122

5 I feel obliged to supervise my child in their community acti 5 0.940

11 My child has friends in and out of school 11 0.866

7 My child has a role model that inspires/encourages them to p 7 0.794

21 It is not difficult to get my child to and from community ac 21 0.641

19 I feel discouraged when searching for community activities t 19 0.488

1 I actively seek opportunities for my child to participate in 1 0.468

10 My child finds it difficult to play with other children 10 0.466

3 My stress levels influence how much I am able to help my chi 3 0.399

2 When necessary I educate supervising adults on how to includ 2 0.388

34 It is hard for my child to participate in cmmty act bo behav 34 0.297

14 I don’t need to travel long way to get to community activiti 14 0.293

36 My child is motivated to participate in activities in the co 36 0.239

37 My family is motivated to participate in the community 37 0.148

33 My child finds it hard to participate in cmmty bo difficulty 33 0.139

31 My child finds it hard to participate in the cmmty bo commun 31 0.137

15 I feel less isolated when my child is involved in community 15 0.105

9 Adults supervising activities in the cmmty make an effrt to 9 -0.047

32 Health professionals have helped/encouraged my child to part 32 -0.112

22 Community activities suitable for my child are within my fa 22 -0.117

27 My child is easily able to participate in community activiti 27 -0.144

29 My child has enough energy to complete community activities 29 -0.396

6 I have enough time to help my child participate in the commu 6 -0.569

13 Mainstream community activity programs include my child 13 -0.602

26 Community activities suitable for my child have long wait li 26 -0.643

16 My child does not enjoy learning new skills and/or is not pe 16 -0.666

20 It is not important for my child to participate in the commu 20 -0.759

35 My child doesn’t want to participate in community activities 35 -0.827

4 My child receives enough funding to cover their community ac 4 -1.103

24 There are enough activity programs in the community that mee 24 -1.272

23 Community activities suitable for my child are advertised wi 23 -1.810

28 My child’s school organises out-of-school events that are in 28 -2.283

12 People in the community understand my child’s ABI, they are 12 -2.611

PQMethod2.35 ABIandcommunityparticipation PAGE 12

Path and Project Name: C:\PQMETHOD\projects/abione 2/14 20

Descending Array of Differences Between Factors 1 and 2

No. Statement No. Type 1 Type 2 Difference

11 My child has friends in and out of school 11 2.039 -0.310 2.349

21 It is not difficult to get my child to and from community ac 21 0.537 -1.404 1.941

22 Community activities suitable for my child are within my fa 22 0.539 -1.269 1.808

6 I have enough time to help my child participate in the commu 6 0.731 -0.890 1.621

27 My child is easily able to participate in community activiti 27 1.148 -0.465 1.613

37 My family is motivated to participate in the community 37 0.660 -0.555 1.214

32 Health professionals have helped/encouraged my child to part 32 0.771 -0.253 1.024

12 People in the community understand my child’s ABI, they are 12 0.507 -0.369 0.876

4 My child receives enough funding to cover their community ac 4 -0.572 -1.421 0.849

14 I don’t need to travel long way to get to community activiti 14 0.261 -0.576 0.837

35 My child doesn’t want to participate in community activities 35 -1.256 -2.056 0.799

24 There are enough activity programs in the community that mee 24 -0.101 -0.679 0.578

7 My child has a role model that inspires/encourages them to p 7 0.317 -0.018 0.336

1 I actively seek opportunities for my child to participate in 1 0.656 0.443 0.214

29 My child has enough energy to complete community activities 29 0.964 0.780 0.184

13 Mainstream community activity programs include my child 13 0.367 0.212 0.154

30 My child finds it hard to participate in the cmmty bo mobili 30 -1.003 -1.119 0.116

36 My child is motivated to participate in activities in the co 36 0.884 0.921 -0.037

16 My child does not enjoy learning new skills and/or is not pe 16 -1.270 -1.168 -0.102

9 Adults supervising activities in the cmmty make an effrt to 9 0.770 0.883 -0.113

26 Community activities suitable for my child have long wait li 26 -0.588 -0.264 -0.323

20 It is not important for my child to participate in the commu 20 -1.773 -1.440 -0.333

15 I feel less isolated when my child is involved in community 15 0.186 0.520 -0.334

25 It makes me happy when my child enjoys participating in com 25 1.904 2.292 -0.388

23 Community activities suitable for my child are advertised wi 23 -0.615 -0.210 -0.406

28 My child’s school organises out-of-school events that are in 28 -0.599 -0.161 -0.438

17 My child is proud of participating in community activities t 17 1.221 1.693 -0.472

8 My child gets frustrated when he/she is not able to do what 8 -0.485 0.024 -0.509

5 I feel obliged to supervise my child in their community acti 5 -0.140 0.679 -0.819

18 My child wants to do what his/her friends and/or siblings do 18 0.980 1.956 -0.976

34 It is hard for my child to participate in cmmty act bo behav 34 -1.125 -0.103 -1.022

2 When necessary I educate supervising adults on how to includ 2 -0.201 1.043 -1.244

3 My stress levels influence how much I am able to help my chi 3 0.032 1.287 -1.255

33 My child finds it hard to participate in cmmty bo difficulty 33 -1.069 0.564 -1.633

19 I feel discouraged when searching for community activities t 19 -1.215 0.515 -1.730

31 My child finds it hard to participate in the cmmty bo commun 31 -1.601 0.346 -1.946

10 My child finds it difficult to play with other children 10 -1.863 0.571 -2.434

PQMethod2.35 ABIandcommunityparticipation PAGE 13

Path and Project Name: C:\PQMETHOD\projects/abione 2/14 20

Descending Array of Differences Between Factors 1 and 3

No. Statement No. Type 1 Type 3 Difference

12 People in the community understand my child’s ABI, they are 12 0.507 -2.611 3.119

28 My child’s school organises out-of-school events that are in 28 -0.599 -2.283 1.684

29 My child has enough energy to complete community activities 29 0.964 -0.396 1.360

6 I have enough time to help my child participate in the commu 6 0.731 -0.569 1.300

27 My child is easily able to participate in community activiti 27 1.148 -0.144 1.292

23 Community activities suitable for my child are advertised wi 23 -0.615 -1.810 1.195

11 My child has friends in and out of school 11 2.039 0.866 1.173

24 There are enough activity programs in the community that mee 24 -0.101 -1.272 1.171

13 Mainstream community activity programs include my child 13 0.367 -0.602 0.969

32 Health professionals have helped/encouraged my child to part 32 0.771 -0.112 0.883

9 Adults supervising activities in the cmmty make an effrt to 9 0.770 -0.047 0.817

22 Community activities suitable for my child are within my fa 22 0.539 -0.117 0.656

36 My child is motivated to participate in activities in the co 36 0.884 0.239 0.645

4 My child receives enough funding to cover their community ac 4 -0.572 -1.103 0.531

37 My family is motivated to participate in the community 37 0.660 0.148 0.511

25 It makes me happy when my child enjoys participating in com 25 1.904 1.709 0.195

1 I actively seek opportunities for my child to participate in 1 0.656 0.468 0.188

15 I feel less isolated when my child is involved in community 15 0.186 0.105 0.081

26 Community activities suitable for my child have long wait li 26 -0.588 -0.643 0.055

14 I don’t need to travel long way to get to community activiti 14 0.261 0.293 -0.031

21 It is not difficult to get my child to and from community ac 21 0.537 0.641 -0.104

18 My child wants to do what his/her friends and/or siblings do 18 0.980 1.122 -0.142

3 My stress levels influence how much I am able to help my chi 3 0.032 0.399 -0.367

35 My child doesn’t want to participate in community activities 35 -1.256 -0.827 -0.430

7 My child has a role model that inspires/encourages them to p 7 0.317 0.794 -0.477

2 When necessary I educate supervising adults on how to includ 2 -0.201 0.388 -0.588

16 My child does not enjoy learning new skills and/or is not pe 16 -1.270 -0.666 -0.604

17 My child is proud of participating in community activities t 17 1.221 2.002 -0.781

20 It is not important for my child to participate in the commu 20 -1.773 -0.759 -1.014

5 I feel obliged to supervise my child in their community acti 5 -0.140 0.940 -1.080

33 My child finds it hard to participate in cmmty bo difficulty 33 -1.069 0.139 -1.208

34 It is hard for my child to participate in cmmty act bo behav 34 -1.125 0.297 -1.422

8 My child gets frustrated when he/she is not able to do what 8 -0.485 1.200 -1.686

19 I feel discouraged when searching for community activities t 19 -1.215 0.488 -1.703

31 My child finds it hard to participate in the cmmty bo commun 31 -1.601 0.137 -1.737

30 My child finds it hard to participate in the cmmty bo mobili 30 -1.003 1.123 -2.125

10 My child finds it difficult to play with other children 10 -1.863 0.466 -2.329

PQMethod2.35 ABIandcommunityparticipation PAGE 14

Path and Project Name: C:\PQMETHOD\projects/abione 2/14 20

Descending Array of Differences Between Factors 2 and 3

No. Statement No. Type 2 Type 3 Difference

12 People in the community understand my child’s ABI, they are 12 -0.369 -2.611 2.242

28 My child’s school organises out-of-school events that are in 28 -0.161 -2.283 2.122

23 Community activities suitable for my child are advertised wi 23 -0.210 -1.810 1.601

29 My child has enough energy to complete community activities 29 0.780 -0.396 1.176

9 Adults supervising activities in the cmmty make an effrt to 9 0.883 -0.047 0.930

3 My stress levels influence how much I am able to help my chi 3 1.287 0.399 0.888

18 My child wants to do what his/her friends and/or siblings do 18 1.956 1.122 0.834

13 Mainstream community activity programs include my child 13 0.212 -0.602 0.815

36 My child is motivated to participate in activities in the co 36 0.921 0.239 0.681

2 When necessary I educate supervising adults on how to includ 2 1.043 0.388 0.656

24 There are enough activity programs in the community that mee 24 -0.679 -1.272 0.593

25 It makes me happy when my child enjoys participating in com 25 2.292 1.709 0.583

33 My child finds it hard to participate in cmmty bo difficulty 33 0.564 0.139 0.425

15 I feel less isolated when my child is involved in community 15 0.520 0.105 0.415

26 Community activities suitable for my child have long wait li 26 -0.264 -0.643 0.379

31 My child finds it hard to participate in the cmmty bo commun 31 0.346 0.137 0.209

10 My child finds it difficult to play with other children 10 0.571 0.466 0.105

19 I feel discouraged when searching for community activities t 19 0.515 0.488 0.028

1 I actively seek opportunities for my child to participate in 1 0.443 0.468 -0.026

32 Health professionals have helped/encouraged my child to part 32 -0.253 -0.112 -0.141

5 I feel obliged to supervise my child in their community acti 5 0.679 0.940 -0.261

17 My child is proud of participating in community activities t 17 1.693 2.002 -0.309

4 My child receives enough funding to cover their community ac 4 -1.421 -1.103 -0.318

6 I have enough time to help my child participate in the commu 6 -0.890 -0.569 -0.320

27 My child is easily able to participate in community activiti 27 -0.465 -0.144 -0.321

34 It is hard for my child to participate in cmmty act bo behav 34 -0.103 0.297 -0.399

16 My child does not enjoy learning new skills and/or is not pe 16 -1.168 -0.666 -0.502

20 It is not important for my child to participate in the commu 20 -1.440 -0.759 -0.681

37 My family is motivated to participate in the community 37 -0.555 0.148 -0.703

7 My child has a role model that inspires/encourages them to p 7 -0.018 0.794 -0.812

14 I don’t need to travel long way to get to community activiti 14 -0.576 0.293 -0.869

22 Community activities suitable for my child are within my fa 22 -1.269 -0.117 -1.152

11 My child has friends in and out of school 11 -0.310 0.866 -1.176

8 My child gets frustrated when he/she is not able to do what 8 0.024 1.200 -1.176

35 My child doesn’t want to participate in community activities 35 -2.056 -0.827 -1.229

21 It is not difficult to get my child to and from community ac 21 -1.404 0.641 -2.045

30 My child finds it hard to participate in the cmmty bo mobili 30 -1.119 1.123 -2.242

PQMethod2.35 ABIandcommunityparticipation PAGE 15

Path and Project Name: C:\PQMETHOD\projects/abione 2/14 20

Factor Q-Sort Values for Each Statement

Factor Arrays

No. Statement No. 1 2 3

1 I actively seek opportunities for my child to participate in 1 1 1 1

2 When necessary I educate supervising adults on how to includ 2 -1 3 1

3 My stress levels influence how much I am able to help my chi 3 0 3 1

4 My child receives enough funding to cover their community ac 4 -1 -4 -3

5 I feel obliged to supervise my child in their community acti 5 0 2 3

6 I have enough time to help my child participate in the commu 6 2 -2 -1

7 My child has a role model that inspires/encourages them to p 7 0 0 2

8 My child gets frustrated when he/she is not able to do what 8 -1 0 4

9 Adults supervising activities in the cmmty make an effrt to 9 2 2 0

10 My child finds it difficult to play with other children 10 -5 2 1

11 My child has friends in and out of school 11 5 -1 2

12 People in the community understand my child’s ABI, they are 12 1 -1 -5

13 Mainstream community activity programs include my child 13 0 0 -2

14 I don’t need to travel long way to get to community activiti 14 0 -2 0

15 I feel less isolated when my child is involved in community 15 0 1 0

16 My child does not enjoy learning new skills and/or is not pe 16 -3 -3 -2

17 My child is proud of participating in community activities t 17 4 4 5

18 My child wants to do what his/her friends and/or siblings do 18 3 4 3

19 I feel discouraged when searching for community activities t 19 -3 1 2

20 It is not important for my child to participate in the commu 20 -4 -4 -2

21 It is not difficult to get my child to and from community ac 21 1 -3 2

22 Community activities suitable for my child are within my fa 22 1 -3 -1

23 Community activities suitable for my child are advertised wi 23 -2 0 -4

24 There are enough activity programs in the community that mee 24 0 -2 -3

25 It makes me happy when my child enjoys participating in com 25 4 5 4

26 Community activities suitable for my child have long wait li 26 -1 -1 -2

27 My child is easily able to participate in community activiti 27 3 -1 -1

28 My child’s school organises out-of-school events that are in 28 -1 0 -4

29 My child has enough energy to complete community activities 29 3 2 -1

30 My child finds it hard to participate in the cmmty bo mobili 30 -2 -2 3

31 My child finds it hard to participate in the cmmty bo commun 31 -4 1 0

32 Health professionals have helped/encouraged my child to part 32 2 0 -1

33 My child finds it hard to participate in cmmty bo difficulty 33 -2 1 0

34 It is hard for my child to participate in cmmty act bo behav 34 -2 0 1

35 My child doesn’t want to participate in community activities 35 -3 -5 -3

36 My child is motivated to participate in activities in the co 36 2 3 0

37 My family is motivated to participate in the community 37 1 -1 0

Variance = 5.676 St. Dev. = 2.382

PQMethod2.35 ABIandcommunityparticipation PAGE 16

Path and Project Name: C:\PQMETHOD\projects/abione 2/14 20

Factor Q-Sort Values for Statements sorted by Consensus vs. Disagreement (Variance across Factor Z-Scores)

Factor Arrays

No. Statement No. 1 2 3

1 I actively seek opportunities for my child to participate in 1 1 1 1

26 Community activities suitable for my child have long wait li 26 -1 -1 -2

15 I feel less isolated when my child is involved in community 15 0 1 0

25 It makes me happy when my child enjoys participating in com 25 4 5 4

16 My child does not enjoy learning new skills and/or is not pe 16 -3 -3 -2

36 My child is motivated to participate in activities in the co 36 2 3 0

17 My child is proud of participating in community activities t 17 4 4 5

7 My child has a role model that inspires/encourages them to p 7 0 0 2

4 My child receives enough funding to cover their community ac 4 -1 -4 -3

14 I don’t need to travel long way to get to community activiti 14 0 -2 0

9 Adults supervising activities in the cmmty make an effrt to 9 2 2 0

20 It is not important for my child to participate in the commu 20 -4 -4 -2

13 Mainstream community activity programs include my child 13 0 0 -2

18 My child wants to do what his/her friends and/or siblings do 18 3 4 3

32 Health professionals have helped/encouraged my child to part 32 2 0 -1

5 I feel obliged to supervise my child in their community acti 5 0 2 3

24 There are enough activity programs in the community that mee 24 0 -2 -3

37 My family is motivated to participate in the community 37 1 -1 0

2 When necessary I educate supervising adults on how to includ 2 -1 3 1

35 My child doesn’t want to participate in community activities 35 -3 -5 -3

3 My stress levels influence how much I am able to help my chi 3 0 3 1

34 It is hard for my child to participate in cmmty act bo behav 34 -2 0 1

29 My child has enough energy to complete community activities 29 3 2 -1

23 Community activities suitable for my child are advertised wi 23 -2 0 -4

33 My child finds it hard to participate in cmmty bo difficulty 33 -2 1 0

27 My child is easily able to participate in community activiti 27 3 -1 -1

6 I have enough time to help my child participate in the commu 6 2 -2 -1

8 My child gets frustrated when he/she is not able to do what 8 -1 0 4

22 Community activities suitable for my child are within my fa 22 1 -3 -1

19 I feel discouraged when searching for community activities t 19 -3 1 2

31 My child finds it hard to participate in the cmmty bo commun 31 -4 1 0

28 My child’s school organises out-of-school events that are in 28 -1 0 -4

21 It is not difficult to get my child to and from community ac 21 1 -3 2

11 My child has friends in and out of school 11 5 -1 2

30 My child finds it hard to participate in the cmmty bo mobili 30 -2 -2 3

10 My child finds it difficult to play with other children 10 -5 2 1

12 People in the community understand my child’s ABI, they are 12 1 -1 -5

PQMethod2.35 ABIandcommunityparticipation PAGE 17

Path and Project Name: C:\PQMETHOD\projects/abione 2/14 20

Factor Characteristics

Factors

1 2 3

No. of Defining Variables 20 7 4

Average Rel. Coef. 0.800 0.800 0.800

Composite Reliability 0.988 0.966 0.941

S.E. of Factor Z-Scores 0.111 0.186 0.243

Standard Errors for Differences in Factor Z-Scores

(Diagonal Entries Are S.E. Within Factors)

Factors 1 2 3

1 0.157 0.216 0.267

2 0.216 0.263 0.305

3 0.267 0.305 0.343

PQMethod2.35 ABIandcommunityparticipation PAGE 18

Path and Project Name: C:\PQMETHOD\projects/abione 2/14 20

Distinguishing Statements for Factor 1

(P < .05 ; Asterisk (*) Indicates Significance at P < .01)

Both the Factor Q-Sort Value (Q-SV) and the Z-Score (Z-SCR) are Shown.

Factors

1 2 3

No. Statement No. Q-SV Z-SCR Q-SV Z-SCR Q-SV Z-SCR

11 My child has friends in and out of school 11 5 2.04* -1 -0.31 2 0.87

17 My child is proud of participating in community activities t 17 4 1.22 4 1.69 5 2.00

27 My child is easily able to participate in community activiti 27 3 1.15* -1 -0.46 -1 -0.14

32 Health professionals have helped/encouraged my child to part 32 2 0.77* 0 -0.25 -1 -0.11

6 I have enough time to help my child participate in the commu 6 2 0.73* -2 -0.89 -1 -0.57

22 Community activities suitable for my child are within my fa 22 1 0.54 -3 -1.27 -1 -0.12

12 People in the community understand my child’s ABI, they are 12 1 0.51* -1 -0.37 -5 -2.61

24 There are enough activity programs in the community that mee 24 0 -0.10* -2 -0.68 -3 -1.27

5 I feel obliged to supervise my child in their community acti 5 0 -0.14* 2 0.68 3 0.94

2 When necessary I educate supervising adults on how to includ 2 -1 -0.20 3 1.04 1 0.39

8 My child gets frustrated when he/she is not able to do what 8 -1 -0.49 0 0.02 4 1.20

4 My child receives enough funding to cover their community ac 4 -1 -0.57 -4 -1.42 -3 -1.10

28 My child’s school organises out-of-school events that are in 28 -1 -0.60 0 -0.16 -4 -2.28

33 My child finds it hard to participate in cmmty bo difficulty 33 -2 -1.07* 1 0.56 0 0.14

34 It is hard for my child to participate in cmmty act bo behav 34 -2 -1.13* 0 -0.10 1 0.30

19 I feel discouraged when searching for community activities t 19 -3 -1.21* 1 0.52 2 0.49

31 My child finds it hard to participate in the cmmty bo commun 31 -4 -1.60* 1 0.35 0 0.14

10 My child finds it difficult to play with other children 10 -5 -1.86* 2 0.57 1 0.47

PQMethod2.35 ABIandcommunityparticipation PAGE 19

Path and Project Name: C:\PQMETHOD\projects/abione 2/14 20

Distinguishing Statements for Factor 2

(P < .05 ; Asterisk (*) Indicates Significance at P < .01)

Both the Factor Q-Sort Value (Q-SV) and the Z-Score (Z-SCR) are Shown.

Factors

1 2 3

No. Statement No. Q-SV Z-SCR Q-SV Z-SCR Q-SV Z-SCR

18 My child wants to do what his/her friends and/or siblings do 18 3 0.98 4 1.96* 3 1.12

3 My stress levels influence how much I am able to help my chi 3 0 0.03 3 1.29* 1 0.40

2 When necessary I educate supervising adults on how to includ 2 -1 -0.20 3 1.04 1 0.39

8 My child gets frustrated when he/she is not able to do what 8 -1 -0.49 0 0.02 4 1.20

28 My child’s school organises out-of-school events that are in 28 -1 -0.60 0 -0.16 -4 -2.28

11 My child has friends in and out of school 11 5 2.04 -1 -0.31* 2 0.87

12 People in the community understand my child’s ABI, they are 12 1 0.51 -1 -0.37* -5 -2.61

37 My family is motivated to participate in the community 37 1 0.66 -1 -0.55 0 0.15

14 I don’t need to travel long way to get to community activiti 14 0 0.26 -2 -0.58* 0 0.29

22 Community activities suitable for my child are within my fa 22 1 0.54 -3 -1.27* -1 -0.12

21 It is not difficult to get my child to and from community ac 21 1 0.54 -3 -1.40* 2 0.64

35 My child doesn’t want to participate in community activities 35 -3 -1.26 -5 -2.06* -3 -0.83

PQMethod2.35 ABIandcommunityparticipation PAGE 20

Path and Project Name: C:\PQMETHOD\projects/abione 2/14 20

Distinguishing Statements for Factor 3

(P < .05 ; Asterisk (*) Indicates Significance at P < .01)

Both the Factor Q-Sort Value (Q-SV) and the Z-Score (Z-SCR) are Shown.

Factors

1 2 3

No. Statement No. Q-SV Z-SCR Q-SV Z-SCR Q-SV Z-SCR

8 My child gets frustrated when he/she is not able to do what 8 -1 -0.49 0 0.02 4 1.20*

30 My child finds it hard to participate in the cmmty bo mobili 30 -2 -1.00 -2 -1.12 3 1.12*

11 My child has friends in and out of school 11 5 2.04 -1 -0.31 2 0.87*

2 When necessary I educate supervising adults on how to includ 2 -1 -0.20 3 1.04 1 0.39

36 My child is motivated to participate in activities in the co 36 2 0.88 3 0.92 0 0.24

9 Adults supervising activities in the cmmty make an effrt to 9 2 0.77 2 0.88 0 -0.05*

22 Community activities suitable for my child are within my fa 22 1 0.54 -3 -1.27 -1 -0.12

29 My child has enough energy to complete community activities 29 3 0.96 2 0.78 -1 -0.40*

13 Mainstream community activity programs include my child 13 0 0.37 0 0.21 -2 -0.60*

20 It is not important for my child to participate in the commu 20 -4 -1.77 -4 -1.44 -2 -0.76

23 Community activities suitable for my child are advertised wi 23 -2 -0.62 0 -0.21 -4 -1.81*

28 My child’s school organises out-of-school events that are in 28 -1 -0.60 0 -0.16 -4 -2.28*

12 People in the community understand my child’s ABI, they are 12 1 0.51 -1 -0.37 -5 -2.61*

PQMethod2.35 ABIandcommunityparticipation PAGE 21

Path and Project Name: C:\PQMETHOD\projects/abione 2/14 20

Consensus Statements -- Those That Do Not Distinguish Between ANY Pair of Factors.

All Listed Statements are Non-Significant at P>.01, and Those Flagged With an * are also Non-Significant at P>.05.

Factors

1 2 3

No. Statement No. Q-SV Z-SCR Q-SV Z-SCR Q-SV Z-SCR

1* I actively seek opportunities for my child to participate in 1 1 0.66 1 0.44 1 0.47

15* I feel less isolated when my child is involved in community 15 0 0.19 1 0.52 0 0.11

16 My child does not enjoy learning new skills and/or is not pe 16 -3 -1.27 -3 -1.17 -2 -0.67

25* It makes me happy when my child enjoys participating in com 25 4 1.90 5 2.29 4 1.71

26* Community activities suitable for my child have long wait li 26 -1 -0.59 -1 -0.26 -2 -0.64

36 My child is motivated to participate in activities in the co 36 2 0.88 3 0.92 0 0.24

QANALYZE was complete at :04:59
